# The Effectiveness of the Capacity Building and Mentorship Program in Improving Evidence-Based Decision-making in the Amhara Region, Northwest Ethiopia: Difference-in-Differences Study

**DOI:** 10.2196/30518

**Published:** 2022-04-22

**Authors:** Moges Asressie Chanyalew, Mezgebu Yitayal, Asmamaw Atnafu, Shegaw Anagaw Mengiste, Binyam Tilahun

**Affiliations:** 1 Department of Health Informatics, Institute of Public Health College of Medicine and Health Sciences University of Gondar Gondar Ethiopia; 2 Department of Health Systems and Policy, Institute of Public Health College of Medicine and Health Sciences University of Gondar Gondar Ethiopia; 3 Management Information Systems School of Business University of South-Eastern Norway Notodden Norway

**Keywords:** capacity building, mentorship, mentoring, mentor, training, data use, information use, facility head, department head, quasi-experiment, difference-in-differences, Ethiopia, Amhara, weak health information system, HIS, health information system, CBMP, DID, decision-making, Africa, evidence based, effectiveness

## Abstract

**Background:**

Weak health information systems (HISs) hobble countries’ abilities to effectively manage and distribute their resources to match the burden of disease. The Capacity Building and Mentorship Program (CBMP) was implemented in select districts of the Amhara region of Ethiopia to improve HIS performance; however, evidence about the effectiveness of the intervention was meager.

**Objective:**

This study aimed to determine the effectiveness of routine health information use for evidence-based decision-making among health facility and department heads in the Amhara region, Northwest Ethiopia.

**Methods:**

The study was conducted in 10 districts of the Amhara region: five were in the intervention group and five were in the comparison group. We employed a quasi-experimental study design in the form of a pretest-posttest comparison group. Data were collected from June to July 2020 from the heads of departments and facilities in 36 intervention and 43 comparison facilities. The sample size was calculated using the double population formula, and we recruited 172 participants from each group. We applied a difference-in-differences analysis approach to determine the effectiveness of the intervention. Heterogeneity of program effect among subgroups was assessed using a triple differences method (ie, difference-in-difference-in-differences [DIDID] method). Thus, the β coefficients, 95% CIs, and *P* values were calculated for each parameter, and we determined that the program was effective if the interaction term was significant at *P*<.05.

**Results:**

Data were collected using the endpoint survey from 155 out of 172 (90.1%) participants in the intervention group and 166 out of 172 (96.5%) participants in the comparison group. The average level of information use for the comparison group was 37.3% (95% CI 31.1%-43.6%) at baseline and 43.7% (95% CI 37.9%-49.5%) at study endpoint. The average level of information use for the intervention group was 52.2% (95% CI 46.2%-58.3%) at baseline and 75.8% (95% CI 71.6%-80.0%) at study endpoint. The study indicated that the net program change over time was 17% (95% CI 5%-28%; *P*=.003). The subgroup analysis also indicated that location showed significant program effect heterogeneity, with a DIDID estimate equal to 0.16 (95% CI 0.026-0.29; *P*=.02). However, sex, age, educational level, salary, and experience did not show significant heterogeneity in program effect, with DIDID estimates of 0.046 (95% CI –0.089 to 0.182), –0.002 (95% CI –0.015 to 0.009), –0.055 (95% CI –0.190 to 0.079), –1.63 (95% CI –5.22 to 1.95), and –0.006 (95% CI –0.017 to 0.005), respectively.

**Conclusions:**

The CBMP was effective at enhancing the capacity of study participants in using the routine HIS for decision-making. We noted that urban facilities had benefited more than their counterparts. The intervention has been shown to produce positive outcomes and should be scaled up to be used in other districts. Moreover, the mentorship modalities for rural facilities should be redesigned to maximize the benefits.

**Trial Registration:**

Pan African Clinical Trials Registry PACTR202001559723931; https://tinyurl.com/3j7e5ka5

## Introduction

A health information system (HIS) is an intersection between health care business processes and information systems to deliver better health care services [1]. An effective and integrated HIS is the foundation of a strong health system and provides underpinnings for decision-making [2,3]. It has much to offer in managing health care costs and improving health care quality [4,5]. Effective decision-making to improve public health care essentially depends on the availability of reliable data [6].

Countries have made tremendous efforts to enhance data use practice for patient care and management. For example, a granular ontology model for maternal and child HISs and a national acute care information platform were implemented and improved data analysis skills and policy making in Pakistan [6] and Sri Lanka [7], respectively. On the other hand, timely feedback on health system performance was implemented in sub-Saharan African countries and resulted in enhanced decision-making among leaders [8]. Likewise, a data-driven quality improvement intervention in Mozambique, Rwanda, and Zambia [9], as well as a data use workshop in Zanzibar and the United Republic of Tanzania [10], were implemented and brought a shift from a lack of awareness to collaborative ownership and improved local use of target indicators to drive change, respectively.

In Botswana, a task-shifting initiative (ie, development of a dedicated monitoring and evaluation cadre) was implemented to strengthen monitoring and evaluation and build a sustainable HIS. As a result, the intervention brought increased use of health data for disease surveillance, operational research, and planning purposes [11]. The Feedback and Analytic Comparison Tool in Egypt [12] and the District Health Profile tool in Kenya [13] were implemented as change drivers; they helped health workers to identify gaps and facilitated data-informed decision-making. Moreover, a partnership-mentoring model implemented in the public hospitals of Ethiopia resulted in a 60% improvement of management indicators [14].

Previous work has indicated that training, supervision, a good perceived culture of information use, having standard indicators, competence on routine HIS (RHIS) tasks, technology enhancement along with capacity building activities, and feedback systems were positively associated with routine health information use [15-18]. Despite this, there has been a concern when using this information in strategic decision-making among health workers [19] as well as a concern that the RHIS was unfairly used to enhance evidence-based decision-making [15]. According to Mate et al [20], incomplete, inaccurate, and untimely data have been challenges of data use. Hoxha et al [16] also documented that the technical, organizational, and behavioral attributes of RHIS data remain challenges in health data use.

The investment in health systems infrastructure or training for clinicians and administrators in low- and middle-income countries (LMIC) has been low [21]. Weak HISs hobble the ability of many LMIC to distribute their resources to match the burden of disease [22], and lack of data use is disempowering staff and those seeking to support them from making progress in setting-relevant research and quality improvement [7]. Considering the low level of health data use among health workers, the Amhara Regional Health Bureau (ARHB), in collaboration with the University of Gondar (UoG) in Ethiopia, introduced the Capacity Building and Mentorship Program (CBMP) in 2019. The initiative has been implemented in five selected districts of the region since then. However, information was meager about the effectiveness of the intervention on the use of information in the study area. Therefore, this study aimed to determine the level of health information use among health facilities and department heads in the Amhara region, Northwest Ethiopia.

## Methods

### Study Setting and Design

Baseline data were collected from April to May 2019 in 10 selected districts of the Amhara region: five were in the intervention group and five were in the comparison group. A year later, endpoint data were collected from June to July 2020 in the same districts. Thus, to estimate the effectiveness of the intervention, a quasi-experimental, nonequivalent, control group study design was employed [23].

**Figure 1:**
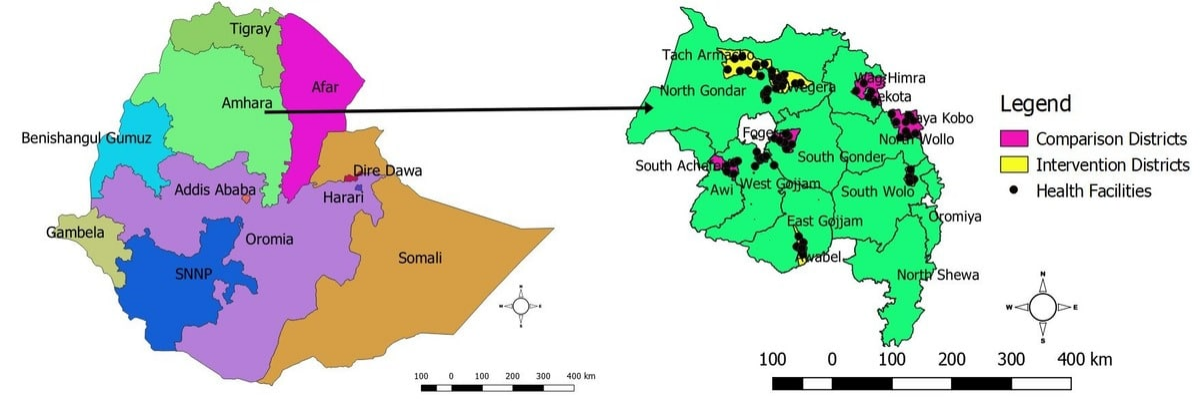
Map of the study area in the Amhara Region, Northwest Ethiopia, 2021.

The Amhara region is located between 8˚45′ N and 13˚45′ N latitude and 35˚46′ E and 40˚25′ E longitude in Northwest Ethiopia [24,25]. It is subdivided administratively into 12 zones and three town administrations (Figure 1). The zones and the town administrations are again subdivided into a total of 189 districts, of which 39 are urban towns. As of 2020, the total population of the region was 22,292,890. The ratio of males to females was close to 1. The region has been implementing a three-tier health system comprised of primary, secondary, and tertiary levels. There are nine referral hospitals, 71 primary hospitals, 954 health centers, and 3450 health posts that are providing health services in the region [26].

### Participants

The source population of this study included all health department and health facility heads. Individuals responsible for the health departments or health institutions that were expected to use routine health data and who were at the selected facilities for at least 6 months were included in the study. However, health department heads who were on leave, retired, and not supposed to use routine health information for patient monitoring and follow-up were excluded from the study. Since it was an intervention effectiveness study, newly inaugurated facilities with patient stays of up to 3 months were also excluded from the study.

### Interventions

The CBMP is an innovative approach that has been implemented since 2019 in selected districts of Ethiopia through the joint venture between the Federal Ministry of Health (FMoH) and selected universities; its goal is to strengthen the national HIS through proper data documentation, information use, and digitalization. The ARHB along with the UoG were responsible for implementing the intervention in the Amhara region. The intervention had two components: tailored training and mentorship. It targeted service delivery unit heads, case team leaders, health department heads, health facility heads, program officers, and district office managers [27].

The intervention was designed primarily to improve the capacity of health workers at different levels. Thus, data quality and information use training manuals were distributed to health facilities, and participants from intervention districts received training on DHIS2 (District Health Information Software 2) data analytics, visualization, presentation, troubleshooting, and data use for decision-making. Furthermore, HIS resources (ie, registers, tally sheets, and computers) were provided to health facilities to enhance the availability and quality of data, and joint review meetings, which served as a platform for discussion, were organized every 6 months [28].

Mentoring is a strategic development activity that supports health workers to attain the vision and goals of the organization [29]. The UoG recruited mentors from departments such as health informatics, health systems and policy, epidemiology and biostatistics, and health education. Thus, training was organized and provided to mentors to introduce them to the HIS strategies being implemented nationally and to provide them with mentorship skills. The trained mentors conducted mentorship programs at each health facility department every quarter for 1 year using the mentorship checklist. As a result, four rounds of mentorship programs were conducted. After that, the mentors with their mentees developed action plans that indicated activities to be accomplished, responsible persons, and implementation period. Based on the mentorship findings, mentees provided detailed written feedback that contained the strengths, weaknesses, and next steps in the performance of the RHIS.

### Outcomes

The dependent variable of the study was the level of information use. The concept of *information used for action in the health care system* was applied. The practice of routine health data use is a process that encompasses gap identification, prioritization, root cause analysis, action plan development, and follow-up. Thus, we used a composite indicator to calculate the average value of information use. The five components of the outcome variable were as follows: identifying indicators in the department, calculating targets versus achievements, providing feedback to health workers at the lower levels, calculating program coverage, and evidence showing the use of data to inform decisions. We calculated the average value of these five indicators and compared the level of information use among intervention and comparison groups [15,30].

### Sample Size

The study employed a quasi-experimental design with pre- and postassessment and intervention and comparison groups. Considering the difference in the level of information use among intervention and comparison districts after the intervention, we employed a double population proportion formula to estimate the sample size. Mathematically, the sample size was determined using the following formula [31]:

*N* = (*K* [*P*_1_ (1 – *P*_1_) + *P*_2_ (1 *– P*_2_)]) / ([*P*_1_ – *P*_2_]^2^)

where N is the sample size; P_1_ is the anticipated proportion of facilities with the attribute of interest (ie, level of information use after the intervention, assuming a 15% increase in information use and considered as 84%) [32]; P_2_ is the proportion of data use with no intervention, taken as 69%; K is the constant at an α value of .05 and a β value of .2, taken as 7.9; and power (1 – β)** **was 80% [33,34]. With these assumptions, the sample size was calculated to be 122 units per department for each group. Though the source population was finite (<10,000), the sample size was corrected using the correction formula. Considering a design effect of 1.5 and a 5% nonresponse rate, the final sample size was 172 for each group. Thus, the overall sample size for both groups combined was 344 individuals.

As a quasi-experimental study, five districts were recruited for each arm (ie, intervention and comparison groups). The intervention districts were selected by the FMoH from among the low-performance districts regarding RHIS activities in the region. However, comparison districts were chosen randomly among the 146 districts in the region. We applied a multistage sampling procedure to select the study participants and developed a sampling frame that contained a list of the heads of departments and facilities in the selected districts. Thus, study participants were selected from the study population using a simple random sampling technique.

### Data Collection Tools and Procedures

Data collection tools were developed based on the Performance of Routine Information System Management tools (version 3) and adapted to the local context [35]. In this paper, we applied the Information Use Assessment Tool and Organizational and Behavioral Assessment Tool (OBAT). The tools were piloted in two districts, Injibara and Debre Tabor, for validity and reliability checks; the districts were out of the study area but comparable with the study sites. The reliability assessment score showed a Cronbach α of .92 for the Likert scale, which indicated that the tool was consistent in measuring the outcome of interest [36].

The Information Use Assessment Tool is an interviewer-administered tool. It was used to examine the health facilities’ report production, information display, discussions, and decisions based on the RHIS, planning, supervision, and mentorship. The OBAT is a self-administered tool that is used to identify information about the technical, organizational, and behavioral constraints for routine health data use. Eight data collectors and two supervisors participated in the data collection. The principal investigator (PI) delivered training on data recording, document review, and ethical consideration to data collectors and supervisors for 2 days. A data quality checklist was developed and applied during data collection to maintain the quality of data. Daily feedback was provided to data collectors by supervisors. The PI led and coordinated the overall data collection process.

Data collectors requested permission from the facility heads to access the documents and departments. In addition, they provided information about the purpose of the study and obtained written consent from selected participants before interviewing them. Following that, they prepared participants for the interview. They reviewed source documents and charts posted in the department or unit, observed the discussion points made among members of the management body in the logbook, and collected data using the study tool. Subsequently, respondents were provided with a self-administered tool (ie, the OBAT) and informed to take an ample amount of time to complete the questionnaire.

### Assignment Methods

The FMoH with the ARHB selected five intervention districts based on predefined criteria. All of these districts were low performers regarding the RHIS activities. They had a low level of information use and poor data quality but could potentially improve their performance if given the intervention. As a result, random assignments of districts to comparison and intervention arms were not applicable. However, the research team, in collaboration with the ARHB, selected five comparison districts to help in measuring the effectiveness of the intervention. Therefore, the intervention groups received usual service and the new CBMP intervention (ie, tailored training and mentorship), and the comparison groups received usual service (ie, supervision and review meeting by routine service).

### Blinding

The nature of the intervention was designed to be implemented by mobile mentors. As a result, we were unable to mask health workers and program implementers. However, all study participants and data collectors were blinded to the research question and hypothesis that the team generated during implementation, baseline, and endpoint data collection.

### Statistical Methods

The team scrutinized the data to identify missing values before entering the data into the software. The data entry template was developed using EpiData software (version 3.1; EpiData Association) by applying the commands and skipping patterns that minimized errors during entry. Thus, cleaned data were entered into EpiData and exported to R software (version 4.0.4; The R Foundation) in CSV file format to compute the effect size of the intervention. R software has different built-in statistical packages that enable researchers to run statistical models and test the hypothesis in question. Descriptive statistics, such as mean and percentage, were calculated. Tables and graphs were also used for presenting findings.

The data were collected from the intervention and comparison districts before and after the implementation of the intervention. Thus, it entailed the difference-in-differences (DID) method to determine the effectiveness of the CBMP. As alluded to by different scholars, the DID method is one of the most frequently used methods in outcome and impact evaluation studies. Based on a combination of comparisons before and after the intervention as well as comparisons of the treatment and comparison groups, the method has an intuitive appeal and has been widely used in economics, public policy, health research, management, and other fields [37,38]. Thus, we employed the DID estimation technique to measure the effectiveness of the intervention using data from before and after the intervention in comparison of intervention and comparison groups. It was applied predominantly to quantify and test whether the level of change in the outcome of interest in the intervention group was significant compared to the comparison group.

The DID approach applied a linear regression model and calculated the change over time in intervention and comparison groups. It double-differenced the change over time in the intervention group compared to the comparison group. The method also generated a valid estimate of the causal effect if the implementation of the CBMP was the only factor that might cause a change in the association between the CBMP and average information use before and after the intervention, as shown in the equation below:

*Y_gt_* = *β*_0_ + *β*_1_*T_g_* + *β*_2_*P_t_* + *β*_3_(*T_g_* × *P_t_*) + *β*_4_(*T_g_ × P_t_ ×* covariates) + *ε_t_*

where Y_gt_ is the average level of information use, β_1_ is the average difference in Y between the two groups that is common in both time periods, β_2_ is the average change in Y from the baseline to the endpoint time period that is common to both groups, β_3_ is the average change in Y from the baseline to the endpoint time period of the intervention group compared to the comparison group, and β_4_ is the triple difference adjusted for some covariates.

To estimate the average change in information use over time using the DID model, we created a dummy variable by assigning 1 to the intervention group and 0 to the comparison group. Moreover, the preintervention period and postintervention period were assigned 0 and 1, respectively. Subsequently, we employed a difference-in-difference-in-differences (DIDID) method to assess whether the program effects were heterogeneous across sex (male vs female), age (≤30 years vs >30 years), educational level (diploma vs above diploma), location (rural vs urban), salary (≤5000 ETB [Ethiopian birr] vs >5000 ETB; a currency exchange rate of 44.32 ETB=US $1 is applicable), and experience (≤5 years vs >5 years) [15].

The model provided information about the β coefficients, *P* values, and 95% CIs. If the coefficient for the interaction term (ie, the DID estimator) was significant at an α value of .05, we determined that the intervention was responsible for causing the change in the treatment group. In addition, the coefficients for the triple difference (ie, the DIDID estimator) were examined using the covariates listed above; one group was judged as having benefited more than the other if the model provided significant β coefficients [37,38].

### Ethics Approval

The study protocol was developed considering the ethical principles from the Declaration of Helsinki. The research protocol was registered at the Pan African Clinical Trials Registry (PACTR202001559723931), which is a World Health Organization International Clinical Trials Registry Platform primary register [39]. Moreover, the registry confirmed that the intervention was implemented with ethical consideration to human subject involvement. The CBMP offers original insights and is a new approach; the tailored intervention was implemented in an adaptive way to address the gaps identified at a specific intervention site. We also secured an ethical clearance letter from the UoG Institutional Review Board (reference No. O/V/P/RCS/05/430/2018). Participants were informed of the purpose of the study and consented before any inquiry. Data were collected anonymously with no personal identifiers; data were used only for this study. We presented findings with no manipulation or subject involvement.

## Results

### Participant Flow Through the Study

**Figure 2:**
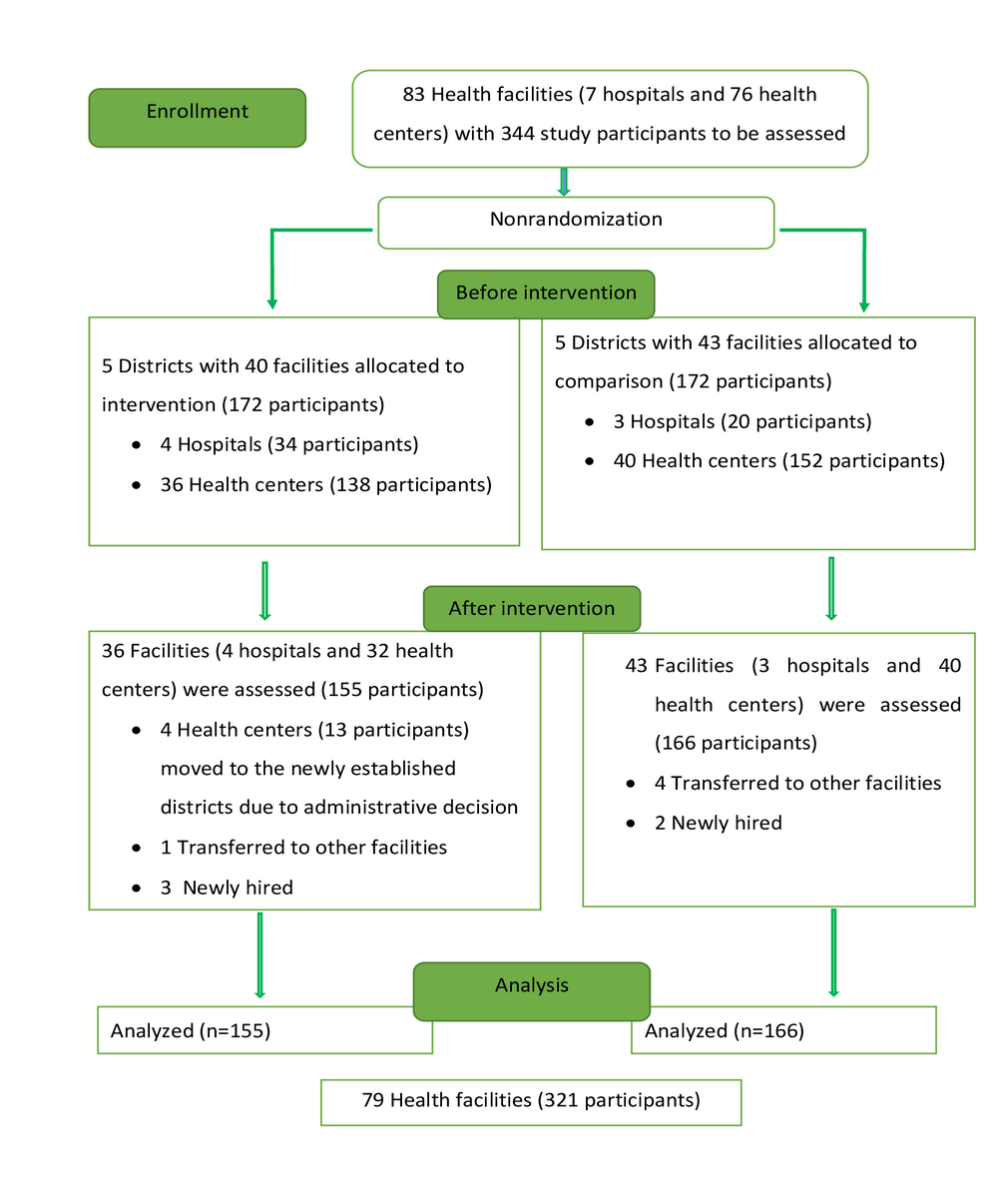
Flow diagram of study participants in the Amhara region, Northwest Ethiopia, 2021.

Baseline data were collected from 344 study participants (intervention: n=172, 50%; comparison: n=172, 50%) across 83 health facilities. However, a total of 321 study participants (intervention: n=155, 48.3%; comparison: n=166, 51.7%) across 79 health facilities (intervention: n=36, 46%; comparison: n=43, 54%) were surveyed at the endpoint of the study; the response rate was 93.3% (321/344). A total of 4 facilities out of 40 (10%) from the intervention arm were excluded because they became part of the newly established districts (Figure 2).

### Characteristics of Study Participants

Among the 321 total study participants, 155 (48.3%) were from the intervention districts and 166 (51.7%) were from the comparison districts. More than half of the study participants were male in both intervention and comparison districts at baseline and endpoint periods. Almost two-thirds of the study participants were below the age of 28 years in both arms. Similarly, more than half of the study participants were diploma holders and resided in rural locations. Two-thirds of the participants earned equal to or below 5000 ETB at baseline, and nearly half of them earned above 5000 ETB at the study endpoint in both the intervention and comparison groups. More than half of the study participants had 5 years or more of experience (Table 1).

**Table 1:** Sociodemographic characteristics of study participants in the Amhara region, Northwest Ethiopia, 2021.

Variable		Baseline							Endpoint				
		Intervention			Comparison			Intervention				Comparison	
		n (%)	95% CI	n (%)		95% CI	n (%)			95% CI	n (%)		95% CI
**Sex**													
	Female	68 (39.5)	32.3-47.3	63 (36.6)		29.5-44.3	68 (43.9)			35.9-52.1	60 (36.4)		29.1-44.2
	Male	104 (60.5)	52.7-67.7	109 (63.4)		55.7-70.5	87 (56.1)			47.9-64.0	106 (63.6)		55.8-70.9
**Age (years)**													
	≤30	133 (77.3)	70.2-83.3	147 (85.5)		79.1-90.2	129 (83.2)			76.2-88.6	130 (78.3)		71.1-84.2
	>30	39 (22.7)	16.8-29.8	25 (14.5)		9.8-20.9	26 (16.8)			11.4-23.8	36 (21.7)		15.8-28.9
**Location**													
	Rural	94 (54.7)	46.9-62.2	122 (70.9)		63.4-77.5	80 (51.6)			43.5-59.7	116 (69.9)		62.2-76.6
	Urban	78 (45.3)	37.8-53.1	50 (29.1)		22.5-36.6	75 (48.4)			40.3-56.5	50 (30.1)		23.4-37.8
**Educational level**													
	Diploma or below	104 (60.5)	52.7-67.7	97 (56.4)		48.6-63.9	87 (56.1)			47.9-64.0	90 (54.5)		46.6-62.2
	Above diploma	68 (39.5)	32.3-47.3	75 (43.6)		36.1-51.4	68 (43.9)			35.9-52.0	75 (45.5)		37.8-53.4
**Salary (ETB^a^)**													
	≤5000	131 (76.2)	68.9-82.2	109 (65.3)		57.5-72.4	77 (49.7)			41.6-54.6	80 (48.5)		40.7-56.4
	>5000	41 (23.8)	17.8-31.0	58 (34.7)		27.6-42.5	78 (50.3)			42.2-58.4	85 (51.5)		43.6-59.3
**Experience (years)**													
	≤5	107 (62.9)	55.2-70.1	128 (65.3)		58.1-71.9	89 (57.4)			49.2-65.2	97 (58.8)		50.9-66.3
	>5	63 (37.1)	29.9-44.8	68 (34.7)		28.1-41.9	66 (42.6)			34.8-50.8	68 (41.2)		33.7-49.1

^a^ETB: Ethiopian birr; a currency exchange rate of 44.32 ETB=US $1 is applicable.

### Component Indicators of Information Use

**Figure 3:**
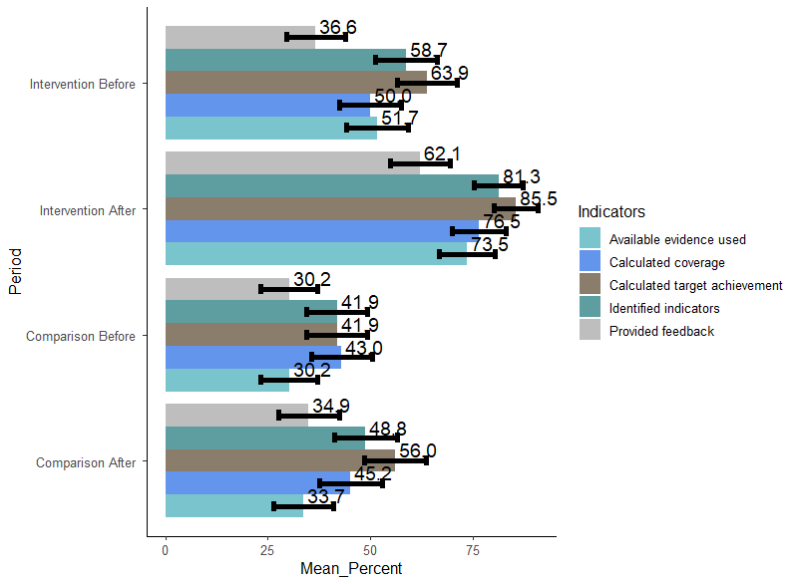
Component indicators of routine information use at baseline and at the study endpoint in the comparison and intervention districts in the Amhara region, Northwest Ethiopia, 2021. The mean values are reported on the bars; the whiskers represent 95% CI values.

The study indicated that a mean of 30.2% (95% CI 23.3-37.2) and 51.7% (95% CI 44.23-59.3) of the department heads in comparison and intervention districts, respectively, used available evidence while making decisions at baseline, whereas a mean of 33.7% (95% CI 26.4-41.0) and 73.5% (95% CI 66.7-80.3) of the department heads in comparison and intervention districts, respectively, used available evidence while making decisions at study endpoint. At baseline, a mean of 41.9% (95% CI 34.5-49.3) and 43.0% (95% CI 35.5-50.5) of the departments in the comparison group calculated target achievement and program coverage, respectively, whereas a mean of 63.9% (95% CI 56.7-71.2) and 50.0% (95% CI 42.5-57.5) of the departments in the intervention group did so. However, the postperiod data showed that a mean of 56.0% (95% CI 48.4-63.7) and 45.2% (95% CI 37.6-52.8) of the comparison groups calculated target achievement and program coverage, respectively, whereas a mean of 85.5% (95% CI 80.1-90.9) and 76.5% (95% CI 70.0-83.0) of the intervention groups did so. At baseline, less than half of the study participants in both groups provided feedback to health workers at the lower levels; however, at the study endpoint, a mean of 34.9% (95% CI 27.5-42.3) of comparison group participants and 62.1% (95% CI 54.6-69.5) of the intervention group participants did so. At baseline, a mean of 41.9% (95% CI 34.5-49.3) and 58.7% (95% CI 51.3-66.2) of the departments in the comparison and intervention groups, respectively, had identified indicators, whereas at the study endpoint, a mean of 48.8% (95% CI 41.1-56.5) and 81.3% (95% CI 75.3-87.3) of the departments in the comparison and intervention groups, respectively, had done so (Figure 3).

### Average Level of Information Use in Decision-making

The average level of information use for the comparison group was 37.3% (95% CI 31.1%-43.6%) at baseline and 43.7% (95% CI 37.9%-49.5%) at the study endpoint. The average level of information use for the intervention group was 52.2% (95% CI 46.2%-58.3%) at baseline and 75.8% (95% CI 71.6%-80.0%) at the study endpoint. The DID analysis indicated that the net program effect change over time was significant (*P*=.003). It indicated that the intervention resulted in a 17% (95% CI 5%-28%) increment in the level of information use among the intervention districts compared to the comparison districts (Table 2).

**Table 2:** DID analysis in control and intervention districts in the Amhara region, Northwest Ethiopia, 2021.

Parameter	Program effect size	95% CI	*P* value
Intercept	0.356	0.300-0.413	<.001
Group (intervention vs comparison)	0.162	0.080-0.243	<.001
Time (study endpoint vs baseline)	0.08	0.000-0.160	.047
DID^a^ analysis (group × time)	0.173	0.058-0.288	.003

^a^DID: difference-in-differences.

### Subgroup Analysis for Program Effect Heterogeneity Diagnosis

The DIDID estimate showed that the CBMP increased information use by 16% among department heads who were working in urban facilities, with a DIDID estimate of 0.16 (95% CI 0.026-0.289; *P*=.02). However, we did not find much evidence of heterogeneity in program effect based on sex, age, educational level, salary, and experience, with DIDID estimates of 0.046 (95% CI –0.089 to 0.182), –0.002 (95% CI –0.015 to 0.009), –0.055 (95% CI –0.190 to 0.079), –1.63 (95% CI –5.22 to 1.95), and –0.006 (95% CI –0.017 to 0.005), respectively (Table 3).

**Table 3:** Subgroup analysis for selected variables in assessing program effect heterogeneity in the Amhara region, Northwest Ethiopia, 2021.

Effect modifier	Heterogeneity in program effect (95% CI)	*P* value
Sex (male vs female )	0.046 (–0.089 to 0.182)	.50
Age (≤30 years vs >30 years)	–0.002 (–0.015 to 0.009)	.65
Educational level (diploma vs above diploma )	–0.055 (–0.190 to 0.079)	.42
Salary (≤5000 ETB^a^ vs >5000 ETB)	–0.00016 (–0.0005 to 0.00019)	.37
Residence (urban vs rural)	0.16 (0.026 to 0.289)	.02
Experience (≤5 years vs >5 years)	–0.006 (–0.017 to 0.005)	.29

^a^ETB: Ethiopian birr; a currency exchange rate of 44.32 ETB=US $1 is applicable.

## Discussion

### Principal Findings

All five component indicators showed high improvement at the study endpoint compared to baseline in each group. The intervention resulted in a 17% change in the average level of information use among study participants in the intervention districts compared to the comparison districts. We noted that the effect of the intervention was heterogeneous in urban and rural facilities. However, significant differences were not observed based on the sex, age, educational level, salary, and experience of the study participants.

This research revealed that the CBMP was effective in improving the capacity of the department and facility heads in using routine health information for decision-making and action. This finding was higher than that found in a cluster randomized controlled trial conducted in Sierra Leone on a community health data review meeting, which resulted in a 14% increment in evidence generation [40]. However, this finding was by far below the findings reported in a study conducted in Nigeria; in that study, data quality and information use training were found to improve feedback mechanisms by 54% [32]. This difference could be due to the large number of facilities covered by the CBMP and the nature of the study participants. Building health workers’ capacity in data use for actions at all levels in the health system would improve and make more efficient the use of health care resources, which would, in turn, lead to making quality services available to clients.

As indicated with these findings, the intervention resulted in the majority of study participants having identified and used indicators to track performance progress in their catchment area. It was consistent with a single study done in Zanzibar and Tanzania where the data use workshop resulted in improvement in the local use of target indicators [10]. Identifying and using indicators in the health system enable health workers to measure the occurrence of disease or other health conditions and factors contributing to them [41]. In addition, indicators link information to actions and provide signals as to whether a program is effective and efficient in achieving the intended results in the target groups [42].

Though the findings highlighted an improvement in providing feedback to health workers at lower levels, it was still unsatisfactory compared to the desired level [27]; however, it was better than the findings of the study done in the Southern Nations, Nationalities, and Peoples Region [43]. It was also inconsistent with the findings obtained in Egypt that reported the effectiveness of the Feedback and Analytic Comparison Tool intervention in improving clinicians’ capacity on providing feedback [12]. The difference might be because the latter was a single intervention primarily targeted at improving feedback mechanisms. In addition, it could be associated with low attention given to the importance of feedback among the study participants in our study. Generating synthesized evidence that indicates the strengths and weaknesses of health workers in the health system is one of the solemn expected activities, among others, in realizing the information revolution agenda [44]. Thus, ineffective feedback mechanisms lead to the provision of poor-quality services to clients and patients.

It was evidenced in a subgroup analysis that urban dwellers benefited more from the intervention than their counterparts. This finding is also in line with the baseline study finding, which indicated that work location was a significant factor associated with the level of information use [15]. This may be because more senior and qualified health workers had transferred from rural to urban facilities. Staff transfer is a common practice in the health system to reduce staff attrition rate [45]. This imbalance created different achievement levels in reaching the information revolution targets in all districts, which, in turn, can affect the quality of care provided to beneficiaries in general.

### Limitations

One limitation of this study was that we did not employ randomization and blinding because of the nature of the study. This may have introduced some information leakage between intervention and comparison districts. Some facilities from which we took baseline data were not included in the endpoint survey, which may have biased the results. Moreover, social desirability and recall bias may also have been introduced, since participants were part of the intervention.

### Conclusions

The CBMP was found to be effective in improving department and facility heads’ capacity in using routine health information for decision-making. The intervention was more beneficial to study participants who resided in urban facilities than their counterparts. A remarkable change was observed in using the available evidence to inform decisions, identify indicators for tracking performance progress, compare targets versus achievements, and calculate program coverage. However, there is still a gap in providing synthesized feedback to health workers at lower levels. Therefore, we propose the following recommendations. The intervention has been proven to produce positive outcomes and should be scaled up to other districts. Moreover, attention should be given to enhance the capacity of health workers employed in rural facilities and to strengthen feedback mechanisms at all levels, in order to reach the desired outcomes of the information revolution.

## Abbreviations

ARHB: Amhara Regional Health Bureau
CBMP: Capacity Building and Mentorship Program
DHIS2: District Health Information Software 2
DID: difference-in-differences
DIDID: difference-in-difference-in-differences
ETB: Ethiopian birr
FMoH: Federal Ministry of Health
HIS: health information system
LMIC: low- and middle-income countries
OBAT: Organizational and Behavioral Assessment Tool
PI: principal investigator
RHIS: routine health information system
UoG: University of Gondar

## Data Availability

Data are available upon reasonable request from the corresponding author.
